# InChagas: Feasibility Study of a Tele-Education Strategy to Promote Early Identification and Care of People Living with Chagas Cardiomyopathy in an Endemic Community in Argentina

**DOI:** 10.5334/gh.1546

**Published:** 2026-04-06

**Authors:** Pablo Elías Gulayin, Christian Gimenez, Daniel Chirino, Bruno Nascimento, Liliana Salvá, Ana Soledad Cavallo, Laura Gutierrez, Laura Blanco, Lorena Paola Cardozo Flores, Eduardo Espina, Saate S. Shakil, Dominique Vervoort, Lindsay Davis, Priscila Raupp, Antonio Luiz Ribeiro, Vilma Irazola

**Affiliations:** 1 Emerging Leaders Program, World Heart Federation, Geneva, Switzerland; 2 Instituto de Efectividad Clínica y Sanitaria (IECS), Buenos Aires, Argentina; 3 Construction Workers Social Security (OSPECON), Franchin Sanatorium, Buenos Aires, Argentina; 4 Serviço de Hemodinâmica, Hospital Madre Teresa, Belo Horizonte, Minas Gerais, Brazil; 5 Departamento de Clínica Médica, Universidade Federal de Minas Gerais, Belo Horizonte, Minas Gerais, Brazil; 6 Jefa de Programa Provincial Control de Enfermedades de Transmisión Vectorial (PPCETV), Ministerio de Salud de San Juan, Argentina; 7 Jefa de División Atención Primaria de la Salud, Ministerio de Salud de San Juan, San Juan, Argentina; 8 Programa Provincial Telesalud, Ministerio de Salud de la provincia de San Juan, San Juan, Argentina; 9 ComitéNacional de Chagas, Federación Argentina de Cardiología, Buenos Aires, Argentina; 10 Programa Provincial Control de Enfermedades de Transmisión Vectorial (PPCETV), Ministerio de Salud de San Juan, Argentina; 11 University of California Los Angeles, Los Angeles, CA, United States; 12 Division of Cardiac Surgery, University of Toronto, Toronto, Ontario, Canada; 13 Foundation “Patients: The Heart of It All”, New York, NY, United States; 14 Novartis Pharma AG, São Paulo, Brazil; 15 Department of Internal Medicine, Faculdade de Medicina, and Telehealth Center and Cardiology Service, Hospital das Clínicas, Universidade Federal de Minas Gerais, Belo Horizonte, Brazil

**Keywords:** Chagas disease, tele-education tools, Chagas cardiomyopathy, primary health care

## Abstract

**Background:**

Chagas disease (ChD) affects over six million people in Latin America, with Chagas cardiomyopathy (CCM) being a severe chronic complication in approximately 30% of those with ChD.

**Objectives:**

To develop, adapt, and evaluate the feasibility, acceptability, and potential impact of a multicomponent tele-education intervention to promote early identification and comprehensive management of CCM among public primary care physicians in an endemic community.

**Methods:**

This mixed-methods study was conducted in San Juan Province, Argentina. The qualitative phase consisted of a needs assessment through local focus groups with health authorities, leaders of local health programs, and healthcare providers from the primary, secondary, and tertiary levels. The quantitative phase, designed as an uncontrolled before-and-after study, involved 23 primary care physicians who participated in a tele-education intervention designed to improve early identification, risk stratification, and appropriate follow-up of CCM, including guidance on the use of the local Tele-Chagas platform. Knowledge, attitudes, and practices (KAP) were assessed through before-and-after intervention surveys. Feasibility and acceptability were evaluated using a 5-point Likert scale.

**Results:**

Focus groups identified limited CCM-specific training as a major barrier to timely referral and treatment. Following the intervention, the median knowledge scores increased from 1 to 2.5 (median difference = 1.5; 95% CI: 0.5–2.5; p = 0.013), and the proportion achieving ≥75% correct answers changed from 0% at baseline to 50% post-intervention. The majority of participants assigned the highest scores (four or five on the Likert scale) to the main characteristics of the educational materials, including weekly delivery, use of WhatsApp as the communication tool, content usefulness, and audiovisual features.

**Conclusions:**

A tele-education intervention demonstrated positive implementation outcomes and significantly improved ChD-related knowledge among participating primary care physicians in San Juan Province, Argentina. Tailored tele-education tools may have the potential to improve CCM management in primary care settings within endemic areas.

## Introduction

According to the World Health Organization, Chagas disease (ChD) affects at least six million people in endemic countries (1,2). South America is one of the main endemic areas, with approximately 25% (~1,500,000) of cases concentrated in Argentina (2,3). During the chronic phase of ChD, severe complications can develop in approximately 30% of infected people. Chagas cardiomyopathy (CCM) is the most common of these complications, associated with high morbidity and economic costs (4,5). Cardiac involvement in ChD is mainly characterized by electrical and mechanical disturbances, including sinus bradycardia, atrial and ventricular arrhythmias, as well as atrioventricular and intraventricular conduction abnormalities (4). Patients may experience clinical complications such as sudden cardiac death, thromboembolism, syncope, or congestive heart failure. Despite this burden, substantial gaps remain in the early diagnosis and management of CCM (6,7,8). The primary care level is an important opportunity to decentralize early detection of these complications and improve linkage to higher-level care for CCM. However, significant barriers hinder the comprehensive clinical management and evaluation of ChD and its complications at this level (9,10). These barriers include lack of systematic case detection, deficiencies in reporting positive cases and their referral for treatment, low coordination between care levels, insufficient evaluation of potential chronic complications, delays in access to medication, and deficiencies in the healthcare team’s knowledge and training regarding updated recommendations for treatment and clinical follow-up of these patients.

In Argentina, the National Ministry of Health published a guideline in 2018 aimed at healthcare teams for the management of patients infected with *Trypanosoma cruzi* (11). This guideline highlights key recommendations for early identification and stratification of cardiac complications. Additionally, in 2020, the World Heart Federation, together with the International Atherosclerosis Society of Cardiology (IASC), published the *Roadmap on Chagas Disease*. This document provides an evidence-based integrated framework for the identification and follow-up of patients with CCM and includes a detailed flowchart to guide patient care across different levels of the healthcare system (4).

In light of the barriers previously mentioned to ensuring quality of care in CCM, the incomplete initial basic evaluation at the primary care level and the late detection of complications of ChD often prevent the early initiation of treatments that could improve morbidity and mortality among infected patients. In this context, despite the availability of general training programs on this neglected disease, the development of a brief and specific package on CCM—tailored to the local primary care level, supported by tele-education tools, and designed to synergize with existing local programs—could contribute to training and updating primary care physicians on a large scale, including those in remote geographic areas.

The objective of this study is to develop, adapt, and evaluate the feasibility, acceptability, and potential impact of a multicomponent tele-education intervention to promote early identification and comprehensive management of CCM among public primary care physicians in the high-risk, endemic province of San Juan, Argentina. We hypothesized that a tailored intervention informed by local stakeholder preferences would improve CCM knowledge among primary care providers in this community.

## Methods

### Study design and participants

A mixed-methods approach was employed, including a qualitative phase focused on a needs assessment and a quantitative phase based on an uncontrolled before-and-after study. This second quasi-experimental phase involved an initial baseline evaluation, the implementation of the designed intervention, and a post-intervention evaluation. This design is especially useful for the evaluation of the effects of educational programs within the context of everyday practice (12).

**Figure 1:**
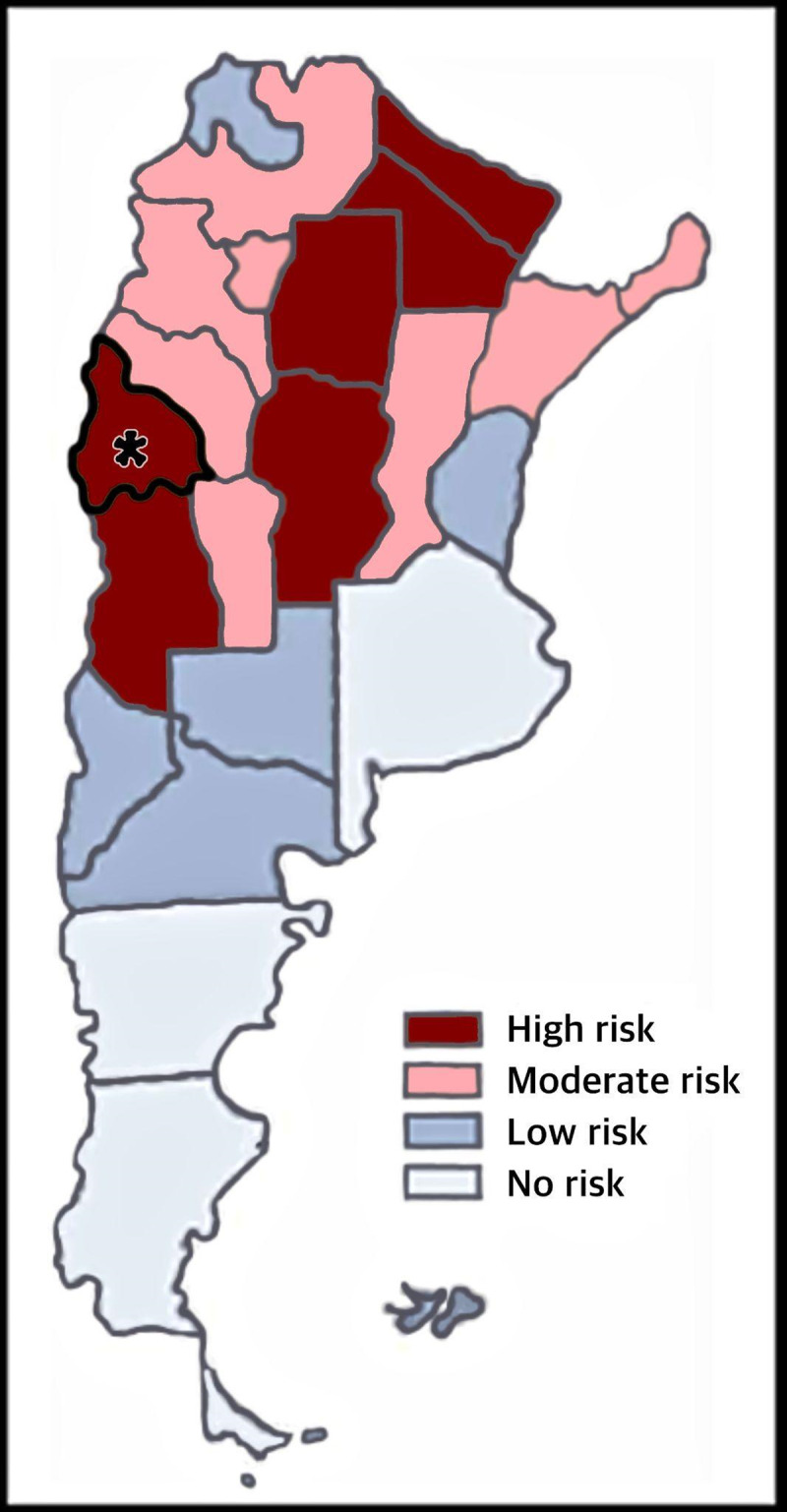
Argentine endemic zones according to vectorial transmission risk. *San Juan Province. Adapted from Spillmann et al. (37).

The study was conducted in the province of San Juan, Argentina, recognized as a high-risk endemic area for ChD transmission (Figure 1). Some areas of this province have among the highest rates of intradomicile infestation in the country (13). With a population of 822,853 inhabitants (14) and 17% living in rural areas (15), the province of San Juan has registered a total of 3,800 seropositive cases in the Argentine Integrated Health Information System (SISA) between 2018 and 2023. The highest number of cases (around 45%) were concentrated in the provincial capital area, where urban household infestation has been documented (16,17). The province of San Juan is divided into five health zones, with approximately 150 adult primary care physicians working in the public health system. Physicians from all these areas were invited to participate in the educational intervention for this study.

As part of the Tele-Health program, the province of San Juan has implemented a web-based tool called Tele-Chagas (18). Currently in the dissemination and early local sensitization stage, this online platform enables health teams to monitor patients undergoing antiparasitic treatment, assess treatment adherence, and coordinate follow-up of the therapeutic regimen and post-treatment evaluations, thereby enhancing communication and coordination between healthcare teams and the provincial ChD program.

### Data collection and measures

#### Qualitative phase

Two focus group sessions were conducted in June 2024. We used a convenient, non-probabilistic sampling method to ensure the involvement of key stakeholders related to the care of patients with ChD in San Juan Province, including health authorities, leaders of local health programs, and healthcare providers from the primary, secondary, and tertiary levels. The development of the interview guide, data collection, and analysis at this phase was based on the Consolidated Framework for Implementation Research (CFIR) (19). Standards for reporting qualitative research (SRQR) (20) guidelines were used to report qualitative results.

During the focus groups, we discussed the following main topics, according to the study objectives: (1) ChD identification, (2) CCM early identification and follow-up, (3) antiparasitic treatment, (4) telemedicine, and (5) training interventions.

The group discussions were facilitated by a trained researcher with expertise in moderating group discussions, who actively encouraged broad participation and ensured that all voices were heard. Sessions were audio-recorded and lasted approximately 80 minutes each. Audio files were anonymized, transcribed verbatim, and manually coded. For the purposes of this publication, selected verbatim segments were translated from Spanish to English. A thematic analysis was conducted manually in accordance with the CFIR, with analytical dimensions developed deductively. Coding was performed by one researcher, and findings were subsequently presented to and discussed with the rest of the research team. An exploratory qualitative approach was used, and thematic saturation was not predefined as a criterion to guide the focus groups. Nevertheless, a recurrence of relevant themes was observed across the focus groups during data analysis. To promote open expression and minimize social desirability bias, participants were informed during the consent process that their contributions were part of a research study, that all data would remain anonymous, that participation was voluntary, and that there were no right or wrong answers.

#### Overview of the before-and-after quantitative phase

To participate in the intervention and receive the educational package, local authorities invited physicians dedicated to adult care in public primary healthcare centers across all health zones of the province. Initially, all physicians received via WhatsApp a brief explanatory video detailing the components of the educational intervention and a link to the SurveyMonkey platform providing access to the baseline survey. Prior to enrollment, all participants reviewed and provided digital informed consent. Each participant was assigned a unique ID, which was used to complete the questionnaires anonymously. The baseline survey was developed following the ‘KAP’ (knowledge, attitudes, and practices) model and considered recommendations from local experts and stakeholders in the field. Table 4 details the questions included in each section of the questionnaire. Specifically, the knowledge-related section (items 1–4) included questions on (1) knowledge of the World Heart Federation’s recommendations for the identification, follow-up, and management of patients with CCM; (2) the most common complications of CCM; (3) the prognosis of electrocardiographic abnormalities in ChD; and (4) the pharmacological treatment of heart failure due to CCM. In addition to the knowledge domain, the KAP questionnaire included items assessing attitudes and self-reported practices related to CCM management. The attitudes domain (items 5–7) explored physicians’ level of agreement with the role of primary care in the initial evaluation of CCM, the need for specific training, and the periodic assessment of asymptomatic infected patients in the primary care setting. The practices domain (items 8–10) assessed self-reported clinical behaviors, including the use of diagnostic tests, referral patterns, and use of the local Tele-Chagas platform to improve patient follow-up. KAP questionnaires are key tools in medical research, particularly in studies evaluating educational interventions or programs, used to assess knowledge, attitudes, and practices related to specific health topics (21). These instruments help identify essential gaps and evaluate changes over time.

Over a period of five weeks following the survey, participating physicians received a remote training program that included a package of brief videos, recorded classes, interactive clinical cases, and an invitation to participate in a webinar with specialists (see Section ‘Intervention’).

After the intervention, the KAP survey was re-administered to participating physicians. In addition, participants completed a follow-up questionnaire to assess implementation outcomes using selected domains of the RE-AIM framework (22). Reach was evaluated through physician recruitment and participation rates, as well as representativeness across the geographic health zones of San Juan Province. Effectiveness, as a proxy for potential impact, was examined through pre–post changes in CCM-related knowledge. Implementation was assessed using early-stage indicators of feasibility and acceptability, including access to and engagement with the educational components (e.g., receipt and viewing of modules) and participant-reported evaluations of content quality, delivery modality, and perceived usefulness, measured using a five-point Likert scale.

The study protocol and the informed consent forms, collected on paper from focus group participants and digitally from physicians participating in the educational intervention, were reviewed and approved by the Provincial Research Ethics Committee (Comité Provincial de Ética en Investigación) of the Ministry of Health of San Juan, Argentina, on 12 April 2024.

### Intervention

The components of the intervention, along with their characteristics and content, are detailed in Table 1 (educational materials were developed in Spanish, with translations or English subtitles prepared for this publication). The intervention design and key educational content were developed and adapted by the research team in collaboration with local health authorities and ChD specialists. The eight educational components were delivered sequentially, following the order presented in Table 1. After an introductory welcome module, the intervention proceeded with a series of components focused on the identification, risk stratification, and appropriate follow-up of CCM. This was followed by a practical guide on the use of the local Tele-Chagas platform and a dedicated module reviewing electrocardiographic abnormalities associated with ChD. Finally, participants received three integrative clinical cases and were invited to attend a live webinar with expert participation addressing the use of antiparasitic treatment in ChD.

**Table 1:** Educational components of the intervention.


EDUCATIONAL COMPONENT ORDER	CHARACTERISTICS/RESOURCES	KEY CONCEPTS

**I. Welcome video**	Brief video of ~1.5 minutes. Available at: https://www.youtube.com/watch?v=CRhU7C8CJF8&list=PLtMRCLDmBFdV8goHaXTokmpZ3g2u4JeYe&index=2	Initial invitation video including the description of the educational intervention components.

**II. Adapted World Heart Federation Chagas Disease algorithm**	Brief video of ~2.5 minutes. Available at: https://www.youtube.com/watch?v=2FHDALEbFEQ&list=PLtMRCLDmBFdV8goHaXTokmpZ3g2u4JeYe&index=3	Animated video on the local adaptation of the World Heart Federation’s Chagas Disease flowchart for CCM evaluation, stratification, and follow-up.

**III. Overview of Chagas disease**	Brief video of ~5 minutes. Available at https://www.youtube.com/watch?v=Vu_i2IuEQpE&list=PLtMRCLDmBFdV8goHaXTokmpZ3g2u4JeYe&index=4	Chagas disease epidemiology, disease progression, and chronic cardiac complications.

**IV. Chagas Cardiomyopathy**	Brief video of ~5 minutes. Available at https://www.youtube.com/watch?v=Lafo9KMuIXo&list=PLtMRCLDmBFdV8goHaXTokmpZ3g2u4JeYe&index=5	Early identification of chronic cardiac complications. Clinical assessment of heart failure in CCM.

**V. Provincial Tele-Chagas Program**	Brief video of ~7 minutes. Available at https://www.youtube.com/watch?v=GF_XCI2SNgU&list=PLtMRCLDmBFdV8goHaXTokmpZ3g2u4JeYe&index=6	Description of the local program and instructions for the correct use of this telemedicine tool.

**VI. Electrocardiographic disorders associated with Chagas disease**	Brief video of ~7 minutes. Available at https://www.youtube.com/watch?v=DLvJcP_4Nbo&list=PLtMRCLDmBFdV8goHaXTokmpZ3g2u4JeYe&index=7	Presentation of the most common EKG disorders in Chagas disease and their classification according to severity.

**VII. Clinical cases**	Three interactive clinical cases. Available at https://view.genially.com/667b411b56c4260014e1bd6b	Presentation of three integrative and interactive clinical cases. This module allows users to navigate through responses, including explanations of the correct answer.

**VIII. Webinar: ‘Antiparasitic Treatment in Chagas Disease: Current Experiences and Future Challenges’**	~50-min duration webinar with the participation of local ministry of health authority in ChD and an expert from a scientific society (Argentine Federation of Cardiology) Link to recorded webinar: https://www.youtube.com/watch?v=c0mg3DxYjPs	Description of local provincial experience in the use of antiparasitic treatment. Analysis of current guidelines recommendations for the use of antiparasitic treatment in ChD and current lines of research in the field.


Abbreviations: ChD: Chagas disease; CCM: Chagas cardiomyopathy; EKG: electrocardiogram.

The qualitative phase, which included two focus group discussions, informed the development of the educational package by (1) confirming the local need for targeted training in CCM adapted to the primary care level; (2) gathering feedback on the current workflow for the assessment and management of patients with positive Chagas serology to assess the feasibility of implementing an adapted WHF flowchart; (3) evaluating the current status of the Tele-Chagas program; and (4) validating the use of biweekly WhatsApp messages as a communication channel.

All information collected at this initial phase helped to define the content and scope of the materials. Classes and all educational materials were finalized by the research team, following the key components of the World Heart Federation Chagas Disease Roadmap (4). The recommended algorithm for cardiovascular evaluation of patients with positive serology was adapted to local needs, expert recommendations, and the guidelines of the Argentine Ministry of Health (11) (English version in Appendix A). In addition, the training program included a module on the local Tele-Chagas program, and one of the educational components consisted of an open webinar discussing the challenges and current evidence for antiparasitic treatment for ChD. During this event, the provincial health ministry presented up-to-date local epidemiological data and their experience in implementing antiparasitic treatments. Furthermore, the Chagas Committee of the Argentine Federation of Cardiology (FAC) summarized the current recommendations and controversies regarding the antiparasitic treatment of ChD oriented to prevent cardiac complications. The Institute for Clinical Effectiveness and Health Policy (IECS) coordinated the event. The webinar recording was emailed to all registered participants.

## Statistical analysis

In the quantitative phase, we describe physicians’ baseline sociodemographic characteristics, prior training, and access to electrocardiogram (EKG). Categorical variables were reported as absolute frequencies and percentages, while continuous variables were summarized using the median and interquartile range (IQR).

For paired before-and-after intervention comparisons, analyses were restricted to participants who completed both assessments (n = 14) in order to evaluate within-participant changes in knowledge. Total knowledge scores, defined as the number of correct answers to the knowledge-related questions (range 0–4), were summarized as medians with IQR and compared using the Wilcoxon signed-rank test, reporting Hodges–Lehmann median differences with 95% confidence intervals (CIs). A two-sided p-value of <0.05 was considered statistically significant.

For the dichotomized knowledge outcome (≥75% correct answers), we report paired proportions before and after the intervention, along with absolute differences and 95% CI.

## Results

### Qualitative phase

A total of 13 healthcare professionals and health authorities participated in the focus groups, 12 of whom were women. Participants held roles across different sectors of the local health system, including primary and secondary care providers, health authorities, and leaders of local health programs. All participants were over 18 years of age and provided written informed consent. The domains and constructs emerging from the analysis are described in Table 2, presenting results according to the perspectives of decision-makers and healthcare providers.

**Table 2:** Qualitative phase summary.


CFIR DOMAIN	CODE	CONCEPT	SUMMARY	VERBATIM

**Outer Setting (Patient needs & resources/External policies & incentives)**	**Detection of Chagas Disease (CD) in general**	Detection, nominalization, serology, and confirmation methods. Barriers and facilitators for CD detection	Detection and nominalization have been strengthened since 2018 with the mandatory registration of cases in the SISA, supported by comprehensive detection strategies in the field and health centers. This is key to improving disease monitoring and control. Barriers such as the centralization of services and lack of access to specialized studies (such as ECKG and echocardiogram) persist, although facilitators like appointment management and free tests within the Provincial Chagas Program aim to improve timely detection and treatment.	“In an endemic province, I think any patient we have in front of us, we consider as potentially chagasic…”

**Inner Setting (Available resources/Networks & communications**	**Detection and initial evaluation of CCM**	Conduct for CCM detection, use of guidelines for CCM management	In some hospitals, there is effective coordination between biochemistry and infectious diseases, allowing for quick and efficient follow-up of Chagas patients. Smaller hospitals face infrastructure limitations, lacking equipment like echocardiograms, which forces the referral of suspected cases to better-equipped centers. In some centers, the initial evaluation includes chest X-ray, electrocardiogram, and clinical examination.	“We have chest X-ray and electro, plus our clinical examination, and from there we refer patients for more in-depth cardiological control, especially if there is any indication, if they have any arrhythmia on the electro, or the patient reports any suspicious symptom, for example, if they have had syncope.”

**Inner Setting (Available resources)**	**Follow-up and referral**	Follow-up and treatment of Heart Failure	Periodic reevaluation is carried out to detect changes or complications over time. Yearly follow-up is recommended for those without abnormal findings or with minor abnormalities. Patients with signs of cardiological abnormalities, such as chagas cardiomyopathy, are referred to a cardiologist for continuous follow-up in collaboration with their primary care physician. Regarding medication for heart failure, when it is not available in a health center, alternatives such as exchange between centers through a WhatsApp group or requests to the provincial program are used, thus ensuring adherence to treatment.	“At the primary level, we always do the follow-up visits, or the control. We send a message and look for the patients to book an appointment.”

**Outer Setting (Patient needs & resources)**	**Antiparasitic treatment in general**	Antiparasitic treatment and barriers to treatment with Benznidazole	Antiparasitic treatment for Chagas disease in San Juan follows the guidelines of the National Ministry of Health, offering treatment to all patients with Chagas in its vertical and acute phase, and to those in the chronic phase without evident cardiomyopathy, to prevent disease progression. Children tend to adhere better to treatment due to fewer side effects. In adults, adverse reactions such as nausea and dizziness can decrease adherence, sometimes leading to treatment suspension and follow-up delays.	“Those who already have evident cardiac involvement, diagnosed by the cardiologist, already have some alteration where antiparasitic treatment would no longer prevent the damage the parasite may cause.”

**Outer Setting (Patient needs & resources)**	**Telemedicine**	Telemedicine and Tele-Chagas	The implementation of telemedicine in San Juan has advanced with tele-assistance, tele-education, and tele-research programs, although barriers such as limited connectivity in some areas and digital literacy persist, hindering full adoption. The national telemedicine strategy aims to improve communication between levels of care and promote the integration of health professionals. Despite initial concerns, technology has proven to be a practical and effective tool, complementing personalized care and general education, with good uptake in programmatic areas. The need for specific audiovisual tools to facilitate its use is highlighted.	“And in relation to Tele-assistance, we are working at the national level with the Tele-Chagas Program, being part of the pilot provinces, along with Santa Fe and other northern provinces, working not only on synchronous tele-assistance but also asynchronous…” “…the platform would be the connection between the secondary, primary and tertiary level, where we have a large gap…” “…this research will reinforce the implementation of the Tele-Chagas strategy…”

**Inner Setting (Access to knowledge & information)**	**Training**	Barriers and facilitators regarding training modalities, training needs in MCC, internet access in the workplace	Every August, the Vector Control Program organizes a provincial symposium on Chagas aimed at the health team, focusing on disease detection and treatment, although with less emphasis on chagas cardiomyopathy. The pandemic boosted the use of digital tools for training, facilitating access in peripheral regions and overcoming transportation barriers. However, there remains a gap in knowledge about Chagas treatment in adults, highlighting the need to train both health professionals and the general population. Despite limited connectivity, technological flexibility has allowed effective use of these tools.	“And actually, when training has been considered, most have focused on disease detection and treatment, but not on cardiomyopathy, at least not in recent years.”


The qualitative phase provided several important insights. In San Juan, the identification of ChD is based on comprehensive strategies combining field surveillance/screening, community education, and telemedicine tools for the follow-up of ChD cases. Despite these efforts, significant barriers remain, such as a lack of knowledge about this health problem among both primary healthcare professionals and the general community, as well as difficulties in accessing specialized services, which limit appropriate follow-up and treatment adherence. A general training on the management of ChD patients is conducted annually, focusing on disease identification and treatment, but with limited emphasis on CCM as a complication and insufficient adaptation to the primary care level. The pandemic has driven an increase in the use of digital tools for training, significantly improving outreach in remote areas. However, all of the participants highlighted an urgent need to train professionals to detect, evaluate, and treat people with ChD and its complications, especially CCM. According to the interviewees, this lack of training hinders the timely referral and treatment of patients.

### Before-and-after quantitative phase

#### Reach

During the recruitment phase, a total of 27 primary care physicians from the public health system were invited to participate in the study. Of these, 23 (85.2%) agreed to participate and completed their sociodemographic information. The study population consisted mainly of women (82.6%), with a median age of 50 years and 22 years of median clinical experience. More than half of the participants (52.2%) were family physicians or general practitioners. More than three out of four reported having access to an EKG at their workplace. With respect to geographical distribution, all five health zones of the province of San Juan were represented. Details of the participants’ baseline sociodemographic characteristics are presented in Table 3. Regarding participation and retention in the educational intervention, the pre-intervention response rate for the KAP questionnaire was 95.6% (22/23), while the post-intervention response rate was 65.2% (15/23). Overall, 14 participants completed both questionnaires and were included in the paired analysis. The sociodemographic characteristics of participants who completed both assessments compared with those who did not are presented in Appendix B; no differences were observed between groups.

**Table 3:** Characteristics of study participants.


SOCIODEMOGRAPHIC CHARACTERISTICS	n = 23

Female sex, % (n)	82.6% (19)

Age, median years (IQR)	50 (38–62)

Years of experience, median years (IQR)	22 (6–38)

**Medical specialty**, % (n)	

Family/general medicine	52.2% (12)

Internal medicine	17.4% (4)

Other	17.4% (4)

No specialty	13.0% (3)

**EKG availability**, % (n)	78.3% (18)

**Health zone**, % (n)	

Health zone 1	17.4% (4)

Health zone 2	13.1% (3)

Health zone 3	21.7% (5)

Health zone 4	26.1% (6)

Health zone 5	21.7% (5)


Abbreviation: EKG: electrocardiogram.

#### Effectiveness

Before-and-after intervention responses to each specific question of the KAP questionnaire are presented in Table 4.

**Table 4:** Results of the before-and-after KAP questionnaire.


**1. Are you aware of the World Heart Federation’s recommendations for the identification, follow-up, and control of patients with Chagas cardiomyopathy**		

	**Before Intervention(n = 22)**	**After Intervention(n = 15)**

Yes	4.5% (1)	60.0% (9)

No	86.4% (19)	40.0% (6)

Don’t know/no answer	9.1% (2)	–

**2. The most common presentations of Chagas cardiomyopathy are thromboembolic events and ventricular aneurysms**		

True	31.8% (7)	42.9% (6)*

False **(correct answer)**	45.4% (10)	57.1% (8)*

Don’t know/no answer	22.8% (5)	–

**3. In a patient with positive Chagas serology, the presence of complete right bundle branch block (CRBBB) and left anterior fascicular block (LAFB) indicates a worse short-term prognosis**		

True	68.1% (15)	33.3% (5)

False **(correct answer)**	27.3% (6)	66.7% (10)

Don’t know/no answer	4.6% (1)	–

**4. Pedro is 60 years old, under joint follow-up with a cardiologist for Chagas disease and heart failure diagnosis. He visits the health center to pick up medication and have his blood pressure checked. In the absence of contraindications, which of the following drug groups should not be missing from his treatment?**		

ACEI/ARB + amiodarone + aspirin	45.4% (10)	13.3% (2)

ACEI/ARB + beta-blockers + aspirin	13.6% (3)	6.7% (1)

ACEI/ARB + beta-blockers + aldosterone antagonists **(correct answer)**	18.2% (4)	66.1% (10)

ACEI/ARB + furosemide + amiodarone	9.1% (2)	–

Don’t know/no answer	13.6% (3)	13.3% (2)

**5. In your opinion, do you believe that the identification and initial evaluation of people with Chagas disease can be done at the primary care level?**		

Strongly disagree	9.1% (2)	6.7% (1)

Somewhat disagree	4.5% (1)	–

Neither agree nor disagree	–	–

Somewhat agree	22.7% (5)	13.3% (2)

Strongly agree	63.6% (14)	80.0% (12)

**6. In your opinion, do you consider that training activities on Chagas disease are necessary for the health team in your workplace?**		

Strongly disagree	9.1% (2)	6.7% (1)

Somewhat disagree	–	–

Neither agree nor disagree	–	–

Somewhat agree	4.5% (1)	6.7% (1)

Strongly agree	86.4% (19)	86.7% (13)

**7. In your opinion, do you think it is important to periodically monitor asymptomatic Chagas disease patients without demonstrated damage at the primary care level?**		

Strongly disagree	9.1% (2)	6.7% (1)

Somewhat disagree	–	–

Neither agree nor disagree	–	–

Somewhat agree	31.8% (7)	–

Strongly agree	59.1% (13)	93.3% (14)

**8. For all individuals from Chagas-endemic areas, I ensure they have had at least one serological test for Chagas in their lifetime**		

Never	–	–

Rarely	–	6.6% (1)

Sometimes	13.6% (3)	–

Often	22.7% (5)	33.3% (5)

Always	63.6% (14)	60.0% (9)

**9. When I receive a patient with positive Chagas serology, I perform a complete physical exam and request an EKG and chest X-ray as complementary methods to assess possible referral to the second level of care**		

Never	–	–

Rarely	–	–

Sometimes	4.5% (1)	–

Often	13.6% (3)	6.7% (1)

Always	81.8% (18)	93.3% (14)

**10. I use the Tele-Chagas platform as a tool to improve the follow-up of my patients with chronic Chagas in the clinic**		

Never	86.4% (19)	53.3% (8)

Rarely	9.1% (2)	26.7% (4)

Sometimes	4.5% (1)	6.7% (1)

Often	–	13.3% (2)

Always	–	–


Values represent % (n). Abbreviations: ACEI/ARB: ACE inhibitors/angiotensin receptor blockers. *(n = 14, 1 missing).

Among participants included in the paired analysis, the median total knowledge score increased from 1 (IQR 0–1) at baseline to 2.5 (IQR 2–3) after the intervention. The Hodges–Lehmann estimate of the paired median difference (post-pre) was +1.5 points (95% CI: 0.5–2.5), indicating a statistically significant improvement in knowledge (Wilcoxon signed-rank test; p = 0.013).

When knowledge was analyzed as a dichotomous outcome (≥75% correct answers), none of the participants met this threshold at baseline (0%, 95% CI: 0–23%), whereas 50% did so after the intervention (95% CI: 23–77%). This corresponds to an absolute increase of 50% (95% CI: 29–79%).

#### Implementation: feasibility and acceptability

After the intervention, 73.9% (17/23) of participants completed the implementation assessment. Overall, feasibility and acceptability indicators were high among participating physicians. Most respondents reported receiving and accessing the majority of the educational components delivered via WhatsApp and rated the materials highly across multiple domains, including delivery format, content relevance, and navigation through clinical cases (Table 5). In addition, a substantial proportion of participants reported that the materials were useful for informing clinical decision-making and had shared them with colleagues not involved in the study. Regarding the webinar, 64.7% (11/17) of participants registered, and 41.2% (7/17) attended the online session. Time constraints and scheduling conflicts were identified as the main barriers to attendance. As the webinar was broadly advertised and open to the general community, a total of 232 individuals registered for the webinar, of whom 127 attended online. More details on the feasibility and acceptability indicators are presented in Table 5.

**Table 5:** Feasibility, utility, and acceptability indicators of the educational intervention.


**Number of modules received (n = 17)**						

None	–					

1 or 2	–					

3 or 4	11.8% (2)					

5 or more	88.2% (15)					

**Videos viewed (n = 17)**						

Watched all	70.6% (12)					

Watched some	29.4% (5)					

Did not watch any	–					

**Level of satisfaction (n = 17)**						

**(1 is poor, 5 is excellent)**	**1**	**2**	**3**	**4**	**5**	Don’t know/no response

Video with adapted animated flowchart from the World Heart Foundation	–	–	–	29.4% (5)	70.6% (12)	–

Class on ‘Epidemiology of Chagas Disease and its Complications’	–	–	–	17.6% (3)	82.4% (14)	–

Class on ‘Comprehensive Approach to Chagas Cardiomyopathy’	–	–	–	11.8% (2)	82.4% (14)	5.9% (1)

Class on ‘Tele-Chagas Program’	–	–	11.8% (2)	23.5% (4)	58.9% (10)	5.9% (1)

Class on ‘Electrocardiographic Disorders’	–	–	11.8% (2)	5.9% (1)	64.7% (11)	17.6% (3)

Webinar on Antiparasitic Treatment in Chagas Disease	–	–	5.9% (1)	11.8% (2)	64.7% (11)	17.6% (3)

Interactive clinical cases	–	–	–	11.8% (2)	70.6% (12)	17.6% (3)

**Training modules characteristics evaluation (n = 17)**						

**(1 is bad and 5 is excellent)**	1	2	3	4	5	Don’t know/no response

Clarity of the content	–	–	5.9% (1)	11.8% (2)	82.4% (14)	–

Weekly delivery of materials	–	–	–	23.5% (4)	76.5% (13)	–

Use of WhatsApp platform for material delivery	–	–	–	11.8% (2)	88.2% (15)	–

Duration of the videos	–	–	–	11.8% (2)	88.2% (15)	–

Audiovisual quality of the videos (animation, image clarity, audio quality)	–	–	5.9% (1)	5.9% (1)	88.2% (15)	–

Topic covered in the webinar	–	–	–	41.2% (7)	41.2% (7)	17.6% (3)

Navigation through the clinical cases	–	–	–	5.9% (1)	76.5% (13)	17.6% (3)

**Potential utility for decision-making (n = 17)**						

Very useful	70.6% (12)					

Quite useful	23.5% (4)					

Will not change my decisions or behaviors	5.9% (1)					

Not very useful	–					

Not useful at all	–					

**Regarding the short videos, was there any video that you couldn’t watch? (n = 17)**						

Yes	17.6% (3)					

No	82.4% (14)					

**What were the reasons you couldn’t watch certain videos? (n = 3)**						

Lack of time	66.7% (2)					

I was not interested in watching the videos	–					

I did not enjoy watching the videos	–					

Lack of memory on my mobile phone	–					

Lack of internet connectivity	33.3% (1)					

**Have you shared the videos with other colleagues? (n = 14, 3 missing values)**						

Yes	64.3% (9)					

No	35.7% (5)					

**Were you able to register for the webinar on antiparasitic treatment organized in collaboration with the Argentine Federation of Cardiology and the government of the province of San Juan? (n = 17)**						

Yes	64.7% (11)					

No	35.3% (6)					

**Were you able to connect live to the webinar? (n = 11)**						

Yes	63.6% (7)					

No	36.4% (4)					

**What was the reason you didn’t register? (n = 6)**						

I didn’t find the chosen topic important	–					

I was not available on that day and time	83.3% (5)					

I don’t like webinars	–					

Others	16.7% (1)					


## Discussion

In this study, we developed a remote educational intervention aimed at primary care physicians to enhance the dissemination of evidence-based recommendations for CCM and evaluated its feasibility and potential impact in a ChD-endemic province of Argentina. The qualitative needs assessment revealed that, although San Juan has made significant efforts to improve ChD identification through integrated strategies and telemedicine tools, barriers such as limited specialized training for healthcare professionals and restricted access to specialist services continue to hinder comprehensive evaluation, effective follow-up, and timely treatment of ChD patients. Notably, despite the annual implementation of general training programs on ChD, a substantial gap remains in ensuring comprehensive care and treatment for CCM.

During the implementation of the educational intervention, the project successfully engaged primary care physicians from the public health system and across all health zones of the province of San Juan, achieving a high response rate among invited physicians (85.2%) and a noteworthy participation rate by the end of the intervention (65.2%). With respect to the impact on the KAP questionnaire, we observed an overall increase in the proportion of correct answers across all knowledge-related questions. Importantly, among participants who completed the pre- and post-intervention assessments, the median score increased by 1.5 points. Moreover, at baseline none of the physicians answered at least 75% of the knowledge items correctly, and by the end of the intervention, this proportion increased to half of the respondents meeting this threshold. Regarding the ‘attitudes’ and ‘practices’ sections, a similar trend was observed, with responses showing greater alignment with the recommendations promoted by the educational materials after the intervention. Furthermore, we observed positive indicators of feasibility, acceptability, and implementation among participants who completed the intervention. These included strong engagement through easy access to the training materials, favorable evaluations of the audiovisual features of the videos and of the tele-educational package overall, and a significant proportion of physicians sharing the received materials with colleagues. In addition, there was considerable registration for the web-based activity (webinar). However, despite the inclusion of the Tele-Chagas platform as part of the educational intervention, its uptake among participating physicians remained limited. As mentioned above, Tele-Chagas is currently in an early dissemination and local sensitization stage, which may have influenced adoption during the study period. This suggests that barriers to adoption are more likely related to structural and organizational factors, such as workflow integration and system-level support, rather than to attitudinal resistance alone. Future iterations of this intervention should therefore incorporate strategies to address these structural barriers, including closer integration with routine clinical workflows and greater efforts to support platform dissemination.

A well-documented and recognized barrier to effective and proper ChD care is the lack of information and training in ChD identification and management among primary care physicians. This knowledge gap contributes to significant underdiagnosis and underreporting of individuals with established cardiomyopathy, particularly at the primary care level (7,8,23,24). Our findings align with previous studies on tele-educational interventions aimed at improving physicians’ knowledge in managing chronic diseases (25) and cardiovascular conditions (26). In the case of ChD, the effectiveness of an online webinar-based educational intervention targeted at healthcare providers, veterinarians, and public health professionals in the United States has been previously assessed. This intervention included a two-hour webinar with expert presentations, and 57 participants completed both pre- and post-assessments on key topics such as transmission, clinical presentation, diagnostics, and treatment. The authors found a statistically significant increase in knowledge in these areas (27). However, tele-educational interventions can vary widely in format, duration, and content components (28). To our knowledge, no published studies have evaluated multicomponent tele-education interventions aimed at improving the identification and follow-up of CCM among primary care physicians in an endemic area.

Our feasibility study provides valuable insights into the potential benefits of these interventions in enhancing primary care physicians’ knowledge of CCM identification and follow-up. The design and content of this tele-educational package could serve as a model for future initiatives in this field, and the approach is adaptable to other contexts and neglected diseases. Importantly, this CCM-focused intervention was designed to complement existing local ChD training activities. As identified in the qualitative phase, annual training initiatives in the province tend to address ChD disease in general, with limited emphasis on CCM and insufficient adaptation to the primary care level. In this context, this tele-education package focuses specifically on CCM and translates national and international recommendations into a primary care–oriented framework, thereby strengthening existing training efforts. Moreover, its modular structure and low-resource digital delivery approach support potential adaptation to other endemic settings, where core components can be preserved while incorporating context-specific content related to local epidemiology, health system organization, and available technological tools. However, further research using more robust study designs is essential to assess the effectiveness of such interventions on clinical outcomes and quality-of-care indicators, which are closely linked to improved patient prognosis. It is important to highlight that, while overall knowledge improved following the intervention, gains were not uniform across all content areas and did not reach an adequate level across all knowledge domains. Specifically, items related to common complications of CCM and the prognosis of electrocardiographic abnormalities showed smaller absolute changes. This pattern suggests that certain content areas may require additional reinforcement or alternative educational strategies, particularly those addressing disease-specific complications or more complex topics, such as the interpretation and prognostic significance of electrocardiographic abnormalities. Several factors may contribute to this, including limited coverage of Chagas in standard health education curricula, competing priorities in resource-constrained health settings, and the persistence of stigma and misinformation surrounding the disease. Moreover, in some regions, the relative invisibility of Chagas compared to other pressing health concerns may reduce awareness and engagement. At the same time, the magnitude of the observed effects should be interpreted with caution, as improvements may partly reflect response bias inherent to self-reported measures and differential attrition between baseline and post-intervention assessments, which could inform the development of targeted strategies to enhance engagement and retention in future studies. In this context, additional strategies could include monitoring module completion, providing timely positive feedback as participants progress, formally integrating this type of training into the orientation of new primary care staff across the province, and offering periodic refresher sessions on key aspects of ChD management.

### Strengths and Limitations

One of the key strengths of this study is that the educational tools were developed in close collaboration with local experts and authorities. This collaborative process helped ensure their relevance and alignment with local practices and standards. Furthermore, the educational materials considered local needs identified during the qualitative phase and were developed in synergy with both international and national recommendations. Specifically, they were designed within the framework of the 2020 World Heart Federation Chagas Disease Roadmap and took into consideration the 2018 national guidelines issued by the Argentine Ministry of Health for the management of patients infected with *Trypanosoma cruzi* infection. Another strength of the study was the use of WhatsApp as a communication channel, due to its widespread accessibility and user-friendly interface. The platform enabled the regular delivery of educational content, facilitating participant engagement and allowing for the implementation of this type of intervention in geographically remote areas. The process indicators related to the use of this tool were positive, and it is worth noting that mHealth tools have also been successfully employed in previous implementation studies with vulnerable populations in Argentina, where mobile phone penetration is high (29,30).

The study also has several limitations. First, it is important to acknowledge that this pilot study used a convenience sampling method. During the qualitative phase, participants represented physicians from different roles within the local healthcare system; however, there remains a possibility that not all perspectives were adequately captured due to sampling bias. As an exploratory qualitative phase, this study relied on focus group discussions; future studies in the field could incorporate in-depth interviews to further explore individual perspectives. During the before-and-after phase, although adult primary care physicians from all local health zones in this endemic province were included in the educational intervention, the sample represents only a limited fraction of the total adult primary care workforce in the province (approximately 150 physicians), which may limit the generalizability of the findings. Another limitation in our pilot study is the low representation of men. The feminization of the healthcare workers in Argentina, particularly in clinical specialties (31,32), together with evidence that female physicians are more likely to adopt online continuing medical education and to participate more actively in voluntary training compared with men, may help explain the gender distribution observed in our study (33,34). In addition, our study focused only on a single province in Argentina; while our locally based and tailored interventions increased provider knowledge in this location, it is possible that the educational materials are not generalizable to other endemic settings. Rather, we view this work as a pilot validation of a methodology for developing tailored educational interventions, which can be adapted elsewhere to generate locally relevant content and delivery strategies. The absence of a control group is another important limitation, as it limits our ability to account for potential confounding variables. Nevertheless, the before-and-after study design is particularly useful for evaluating educational interventions in public health, especially in routine practice and real-world settings (12). The 65% response rate to the post-intervention KAP questionnaire may have introduced non-response bias, thereby threatening our conclusions and their external validity. However, it is well recognized that achieving high response rates among healthcare professionals is particularly challenging, and our study was close to the minimum threshold of 70% response rate suggested in the survey response rate literature in health sciences education research (35,36). Additionally, self-reported data from the KAP questionnaire may be subject to social desirability bias. However, the use of anonymized IDs and de-identified responses likely minimized this risk. Finally, we did not assess the impact of these provider-focused interventions on patient clinical outcomes, such as CCM case identification and referral, optimal heart failure management, and other measures of morbidity and mortality. In the future, multi-site studies that also assess patient outcomes will serve as important validation of both our methodology and interventions.

## Conclusions

This study assessed the feasibility and potential impact of a multicomponent tele-education intervention designed to enhance physicians’ knowledge and promote the dissemination of evidence-based clinical recommendations for the diagnosis and management of CCM at the primary care level. The intervention demonstrated positive implementation outcomes and resulted in a significant increase in ChD-related knowledge among participating primary care physicians in the endemic province of San Juan, Argentina. Further research is needed to evaluate the long-term impact on patient clinical outcomes and generalizability to other geographic settings.

## Data Accessibility Statement

The data that support the findings of this study are available from the corresponding author upon formal request.

## Additional Files

The additional files for this article can be found as follows:

10.5334/gh.1546.s1 Appendix A. Local adaptation of the WHF flowchart.

10.5334/gh.1546.s2 Appendix B. Sociodemographic characteristics of participants who completed both assessments compared with those who did not.
