# Supplementary figures and images for: InChagas: Feasibility Study of a Tele-Education Strategy to Promote Early Identification and Care of People Living with Chagas Cardiomyopathy in an Endemic Community in Argentina
Source: Glob Heart. 2026 Apr 6;21(1):32. doi: 10.5334/gh.1546 (PMC13068087; doi:10.5334/gh.1546)

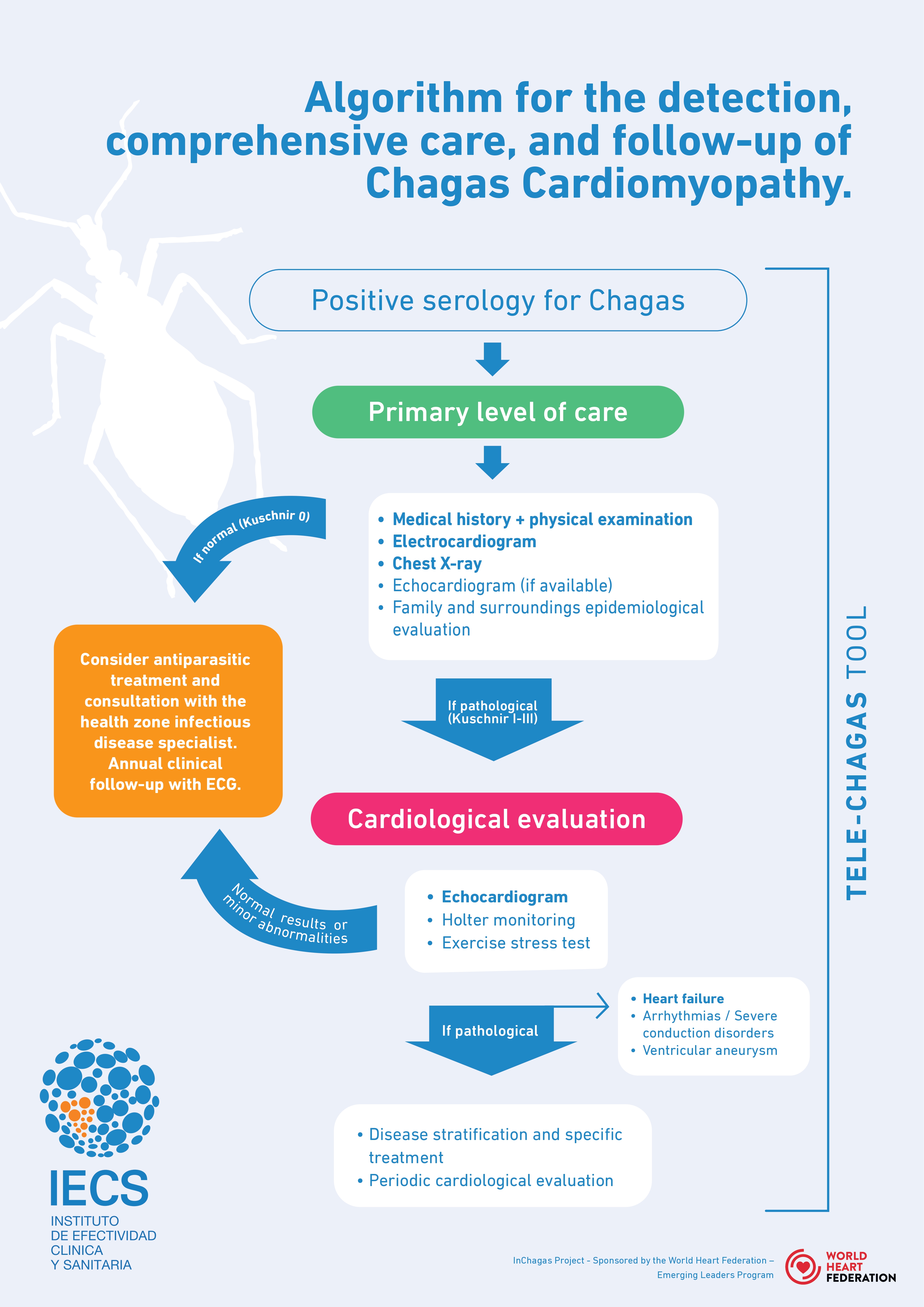

Supplement: Appendix A. — Local adaptation of the WHF flowchart. [file gh-21-1-1546-s1.jpg]
