# Supplementary material for: InChagas: Feasibility Study of a Tele-Education Strategy to Promote Early Identification and Care of People Living with Chagas Cardiomyopathy in an Endemic Community in Argentina
Source: Glob Heart. 2026 Apr 6;21(1):32. doi: 10.5334/gh.1546 (PMC13068087; doi:10.5334/gh.1546)
Supplement: Appendix B. — Sociodemographic characteristics of participants who completed both assessments compared with those who did not. [file gh-21-1-1546-s2.pdf]

**Table 1B: Sociodemographic characteristics of participants who completed both assessments compared with those who did not.**

| | **Paired sample (n = 14)** | **Not paired sample (n = 9)** | **p-value** |
|---|---|---|---|
| Female sex, % (n) | 78.6% (11) | 88.9% (89) | 1.0000 |
| Age, median years (IQR) | 50.5 (44.5 - 53.5) | 47 (42 – 53) | 0.5077 |
| Years of experience, median years (IQR) | 21.5 (16.2 - 26.5) | 22 (10 – 23) | 0.5920 |
| Medical specialty, % (n) | | | 0.4306 |
| Family/general medicine | 42.9% (6) | 66.7% (6) | |
| Internal Medicine | 28.5% (4) | - | |
| Other | 14.3% (2) | 22.2% (2) | |
| No speciality | 14.3% (2) | 11.1% (1) | |

Continuous variables are presented as median (interquartile range) and were compared using the Wilcoxon rank-sum test. Categorical variables are presented as number (percentage) and were compared using Fisher's exact test.
